# The TREM2 paradox in fibrosis: a unified mechanism for opposite outcomes across organs

**DOI:** 10.3389/fimmu.2026.1799143

**Published:** 2026-06-16

**Authors:** Xiuping Liang, Yanhong Li, Ziyi Tang, Guan Wang, Yi Liu

**Affiliations:** 1 Department of Rheumatology & Immunology, Laboratory of Rheumatology and Immunology, West China Hospital, Sichuan University, Chengdu, Sichuan, China; 2 West China Lecheng Hospital, Sichuan University, Boao, Hainan, China

**Keywords:** biomarker, fibrosis, immunometabolism, macrophage polarization, therapeutic target, TREM2

## Abstract

Organ fibrosis, a debilitating outcome of chronic diseases, results from the maladaptive interaction between inflammatory activation and tissue repair mechanisms. Triggering receptor expressed on myeloid cells 2 (TREM2), which is primarily expressed on macrophages, has emerged as a crucial regulator of this process. However, its seemingly contradictory roles across various fibrotic contexts have impeded the development of a cohesive understanding. This review seeks to transcend a simplistic organ-based categorization by proposing that the dual role of TREM2 in fibrosis is dictated by its central function in orchestrating macrophage functional polarization, immunometabolic reprogramming, and multicellular communication within the damaged microenvironment. First, we elucidate the molecular foundations of TREM2 signaling. We subsequently integrate evidence from pulmonary, renal, hepatic, cardiac, and dermal fibrosis to support the premise that TREM2 serves as a pivotal determinant in directing macrophages toward either resolving or perpetuating fibrosis through these fundamental pathways. Furthermore, we critically assessed the translational implications, including the potential of soluble TREM2 (sTREM2) as a dynamic biomarker and the promise of innovative therapeutic approaches.

## 1 Methods

This comprehensive narrative literature review was performed using the PubMed and Google Scholar databases. The selection criteria for the literature were based on content relevance and publication date, with a primary focus on data from the past decade (2015 to the present). Nevertheless, relevance was prioritized over the publication date. Only studies published in English were considered. The search strategy employed a combination of terms, including “TREM2”, “macrophage”, “fibrosis”, “pulmonary fibrosis”, “liver”, “kidney”, “cardiac fibrosis”, and “skin fibrosis”. In PubMed, these terms were explored as both “all fields” and “MeSH” (Medical Subject Headings) terms. Furthermore, additional articles were identified by examining the reference lists of the relevant literature.

## 2 Introduction

Fibrosis is a common feature of many organ diseases, arising from an imbalance caused by ongoing inflammation and faulty tissue repair after cell damage (1, 2). This creates a self-sustaining cycle in which inflammation activates fibroblasts and immune cells, leading to excessive extracellular matrix (ECM) deposition and fibrosis. The fibrotic environment subsequently maintains chronic inflammation, ultimately damaging and impairing organ function (3–5). This inflammation–fibrosis cycle is prevalent in organs such as the heart, lungs, liver, and kidneys (6, 7). Acute kidney injury can cause tissue damage and inflammation, leading to macrophages adopting an anti-inflammatory role to aid fibroblasts in tissue repair. However, ongoing inflammation may excessively activate these cells, resulting in ECM buildup and a cycle of inflammation and fibrosis, ultimately causing renal fibrosis (8–10).

Triggering receptor expressed on myeloid cells (TREM2) is a receptor on myeloid cells, such as macrophages, that regulates inflammation, repair, and fibrosis by responding to tissue signals (11, 12). In inflammation, TREM2 acts as an “inflammatory brake,” reducing inflammation and aiding repair. For example, soluble TREM2 (sTREM2) promotes macrophage M2 polarization, lowering joint inflammation in osteoarthritis (13). However, during the fibrotic process, the function of TREM2 exhibits significant organ specificity. For example, in models of liver fibrosis, TREM2 exhibits antifibrotic effects by enhancing the collagen-degrading capabilities of macrophages and inhibiting the activation of hepatic stellate cells (14, 15). Conversely, in pulmonary fibrosis models, the upregulation of TREM2 expression is positively correlated with the severity of fibrosis, whereas its downregulation may mitigate fibrosis, potentially through the inhibition of the STAT6 signaling pathway (16). This seemingly contradictory role of TREM2 across various fibrotic contexts complicates the establishment of a cohesive understanding of its function.

Therefore, this review seeks to transcend a mere organ-based categorization by systematically investigating the regulatory network of TREM2 in the fibrosis of key organs, including the lungs, kidneys, liver, heart, and skin. Its role in modulating inflammatory responses, macrophage polarization, metabolic reprogramming, and extracellular matrix remodeling, all of which influence fibrosis progression, will be elucidated.

## 3 TREM2: gene, structure, signal transduction and distribution

**Figure 1:**
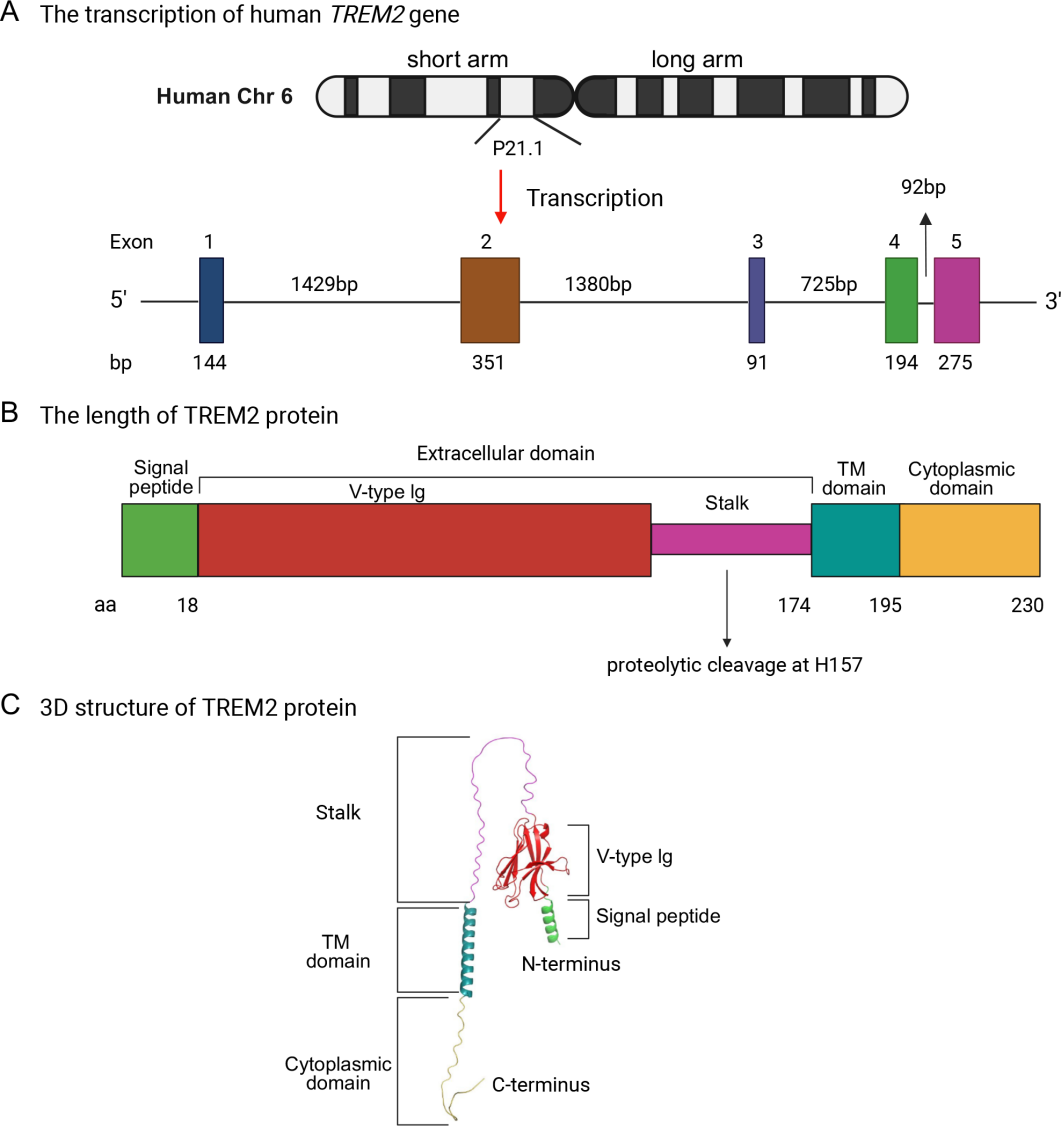
Genomic, transcriptional, and protein features of TREM2. **(A)** Transcription of the human TREM2 gene. **(B)** Structural composition of full-length TREM2. **(C)** 3D structure of the TREM2 protein. The figure was produced using biorender (https://app.biorender.com). TREM2, triggering receptor expressed on myeloid cells; chr, chromosome; TM, transmembrane.

TREM2, which is part of the TREM family and the immunoglobulin superfamily, is encoded by a gene on chromosome 6 (6p21.1) with 5 exons and 4 introns, resulting in the production of a 230-amino acid protein (**Figure 1A**) (17). Its structure is evolutionarily conserved (18, 19). Missense mutations such as R47H can impair TREM2 function, increasing the risk of Alzheimer’s disease (AD) (20). TREM2 includes (1) an extracellular Ig-like domain for ligand binding (21), which is cleaved by ADAM10/17 to form soluble sTREM2 and a C-terminal fragment (TREM2-CTF) (22, 23) (2); a transmembrane domain linked to DNAX-activating protein 12 (DAP12) (24); and (3) a short intracellular domain (ITAM) that relies on DAP12 for signaling (**Figures 1B, C**) (25).

The function of TREM2 relies on signal transduction, where its Ig-like domain uses three complementarity-determining regions (CDR1-3) to recognize ligands, among which the CDR2 loop is pivotal for ligand recognition (26) and binding anionic ligands such as amyloid β (Aβ), apolipoprotein E (ApoE), phosphatidylserine (PS), and heparan sulfate (HS) through hydrophobic and basic interactions (27, 28). Ligand binding triggers conformational changes: PS binding rearranges the CDR2 loop to reveal a positive surface (26), whereas HS binding is stabilized by salt bridges with Arg47 and Arg62 (29). TREM2 and DAP12 form a heterodimer through electrostatic interactions, existing in either a “tightly coupled” or “loosely dissociated” state (24). In the resting state, tight coupling prevents the ITAM motif of DAP12 from being phosphorylated, preventing spleen tyrosine kinase (SYK) kinase recruitment and signaling activation (25, 30). Ligand binding induces helical twisting and conformational changes, exposing and phosphorylating the ITAM, which activates the SYK-PLCγ2-NFAT signaling cascade, promoting phagocytosis, inflammation regulation, and cell survival (31, 32).

TREM2 is highly expressed in specific tissues and is predominantly concentrated in the central nervous system (CNS) (33) and immune-related tissues (34). At the tissue level, TREM2 is widely expressed in the CNS (brain (35) and spinal cord (36)) and immune organs (spleen (37), lymph nodes (38), and bone marrow (39)), with additional distribution in peripheral organs such as the liver (40), lungs (41), kidneys (42), and intestines (43). Its cellular expression is confined primarily to myeloid-derived immune cells. In the CNS, TREM2 is highly expressed specifically in microglia (44), where it regulates their phagocytic functions (35), migration (45), proliferation dynamics (46), and neuroinflammatory responses (47) and plays a critical role in neurodegenerative diseases (48) such as Alzheimer’s disease (44). In peripheral organs, TREM2 is broadly expressed in tissue-resident macrophages within adipose tissue (49), skin (50), intestines (51), liver (52), and alveolar compartments (53), where it mediates phagocytic clearance (54), inflammation, modulation (55), and tissue repair (56). Furthermore, TREM2 in dendritic cells modulates antigen presentation to influence immune responses (57), whereas in osteoclasts, it regulates bone resorption to maintain skeletal homeostasis (58). Its complex distribution highlights the critical role of TREM2 in regulating neuroimmune functions and maintaining organ homeostasis.

## 4 TREM2: the core regulator of macrophage-driven fibrosis

TREM2 profoundly influences the process of organ fibrosis by regulating multiple functional programs of macrophages, including phagocytic clearance, metabolic reprogramming, polarization and inflammatory modulation, cell survival and proliferation, and the secretory profile. These functions are not isolated but interconnected, and their ultimate effect—promoting fibrosis or driving resolution—is highly dependent on the disease stage, organ microenvironment, and ligand repertoire. This highlights its complex and crucial bidirectional regulatory role.

### 4.1 Increased cell survival and proliferation

TREM2 expression significantly increases macrophage viability and proliferation under stress and inflammatory microenvironments by activating key survival signaling pathways, such as the PI3K/AKT/mTOR pathway (58, 59, 79). For instance, in lung fibrosis, this pathway facilitates the prolonged survival of monocyte-derived alveolar macrophages (41). Similarly, in a renal fibrosis model (unilateral ureteral obstruction, UUO), TREM2 plays a role in determining macrophage fate by modulating mTOR-mediated survival signaling (42). This mechanism provides the cytological foundation for the sustained presence and functionality of macrophages within fibrotic lesions.

### 4.2 Mediating immunometabolic reprogramming

As a crucial metabolic sensor, TREM2 plays a significant role in modulating macrophage metabolism to adapt to pathological microenvironments. Its extensive regulatory functions encompass the detection of lipids such as sphingomyelin and oxidized lipoproteins, as well as the reconfiguration of intracellular metabolic pathways, including fatty acid oxidation and the tricarboxylic acid (TCA) cycle (59–61). In fibrotic tissues, the metabolic reprogramming associated with TREM2 is intricately linked to the accumulation or utilization of key immunometabolites. For example, succinate can accumulate under inflammatory conditions, promoting macrophage activation through the stabilization of HIF-1α, thereby enhancing the production of proinflammatory and profibrotic mediators such as IL-1β and TGF-β, which in turn, further amplifies fibroblast activation and extracellular matrix deposition (62, 63). Conversely, itaconate has been implicated in the resolution phase of injury, where it limits succinate dehydrogenase activity, reduces mitochondrial reactive oxygen species (ROS) generation, and suppresses inflammatory signaling, thereby mitigating excessive fibrotic remodeling (64). For instance, TREM2 plays a role in identifying dysregulated sphingolipid metabolism within pulmonary tissue and stimulates the secretion of chemokines (53). Additionally, in the context of post-myocardial infarction repair, it modulates macrophage metabolism towards itaconate production via the SYK/SMAD4 signaling pathway (61). These findings indicate that metabolic reprogramming constitutes a fundamental mechanism by which TREM2 confers functional plasticity to macrophages.

### 4.3 Regulation of phagocytic clearance function

TREM2-dependent phagocytosis is a fundamental mechanism involved in the maintenance of tissue homeostasis, facilitating the clearance of apoptotic cells, protein aggregates, and excess lipids (44, 65). This phagocytic activity is augmented through the activation of downstream signaling pathways, such as the PI3K/AKT pathway (42). In the initial stages of renal fibrosis, as exemplified by the unilateral ureteral obstruction (UUO) model, TREM2 plays a protective role by enhancing the phagocytic capacity of macrophages, thereby enabling the efficient clearance of necrotic debris (42, 66). In contrast, in liver fibrosis, a deficiency in TREM2 function results in inadequate clearance of apoptotic hepatocytes, leading to the accumulation of cellular debris. This accumulation exacerbates inflammation and perpetuates the fibrotic cycle through the release of damage-associated molecular patterns (DAMPs) (15, 54). Consequently, the efficacy of phagocytic function is a critical determinant of the initiation and progression of fibrosis.

### 4.4 Modulating the polarization state and inflammatory balance

The conventional ‘M1/M2’ classification serves as a useful shorthand, it inadequately encapsulates the intricate spatial and pathological stage-specific heterogeneity of macrophages in fibrosis. However, to accurately reflect the cited studies, the original nomenclatures are preserved here, accompanied by specific experimental evidence. TREM2 has a context-dependent, bidirectional regulatory effect on macrophage polarization, which is crucial for its role in determining the direction of fibrosis (profibrotic versus antifibrotic). Its universal mechanisms involve the regulation of key signaling pathways, such as the JAK/STAT and NF-κB pathways (67). In specific models of renal injury, TREM2 mitigates inflammation-induced damage by inhibiting the JAK/STAT pathway, thereby preventing excessive macrophage activation (42, 66). Conversely, in the chronic injury environment of lung fibrosis, TREM2 may facilitate macrophage polarization toward a profibrotic M2-like phenotype through STAT6 signaling (16). This dynamic regulation of polarization exemplifies the adaptive response of TREM2 to varying microenvironmental signals.

### 4.5 Shaping the secretory profile of cytokines and mediators

TREM2 signaling significantly influences the secretory profile of macrophages, functioning as the pivotal step in facilitating intercellular communication and directly impacting fibroblasts and the extracellular matrix. This regulatory mechanism is highly context-dependent. In progressive lung fibrosis, TREM2^+^ macrophages are induced to secrete substantial quantities of TGF-β1, SPP1, and PDGF-A, thereby directly activating fibroblasts and promoting collagen deposition (41). In contrast, during the regression phase of liver fibrosis, TREM2 increases the expression of matrix-degrading enzymes such as MMP9 and MMP12 in macrophages, thereby directly contributing to scar resolution (14, 68). The fundamental variation in secretory products exemplifies distinct downstream outcomes generated by the same set of upstream regulatory mechanisms under varying conditions.

### 4.6 Others

In addition to its role above, TREM2 plays a significant role in tissue remodeling associated with aging and the immunoregulation of T cells. In the aortas of aged mice, there is an upregulation of TREM2 expression in senescence-associated macrophages. Furthermore, IL-13can activate the downstream TREM2–Syk–Sp1–SLC25A51 signaling pathway, facilitating protective interactions between macrophages and vascular smooth muscle cells, thereby mitigating vascular aging (69). Conversely, during the process of fracture healing, there is a reduction in TREM2 expression in macrophages from aged mice. TREM2 deficiency in young mice results in fracture-healing defects akin to those observed with aging, along with an inflammatory imbalance, suggesting that the loss of TREM2 contributes to diminished repair capacity with advancing age (70). Within the central nervous system, microglia with high TREM2 expression are evident in models of aging, amyloidosis, and tauopathy. A deficiency in TREM2 leads to a reduction in microglial numbers and is associated with impaired long-term potentiation (LTP) and postsynaptic loss, underscoring its critical role in neuroaging (71, 72).

In hepatocellular carcinoma driven by non-alcoholic steatohepatitis (NASH), elevated expression of TREM2 is correlated with the infiltration of PD-1^+^Eomes^+^CD8^+^ T cells and regulatory T cells (Tregs). The deletion of Trem2 results in the suppression of TGF-β production via P-Syk-dependent exocytosis, consequently influencing the differentiation of these T cell subsets (73). Overall, these findings suggest that TREM2 not only participates in aging-related tissue remodeling but may also regulate the differentiation of T cells and other immune cells, thereby shaping the immune microenvironment.

In summary, through the core functional modules described above, TREM2 may represent a common molecular framework for regulating macrophage involvement in fibrosis. These mechanisms are themselves conserved across organs. The specific role of TREM2 is the result of the differential recruitment of these universal modules by the local, unique microenvironment.

## 5 Specific integration of TREM2 in organ fibrosis

**Figure 2:**
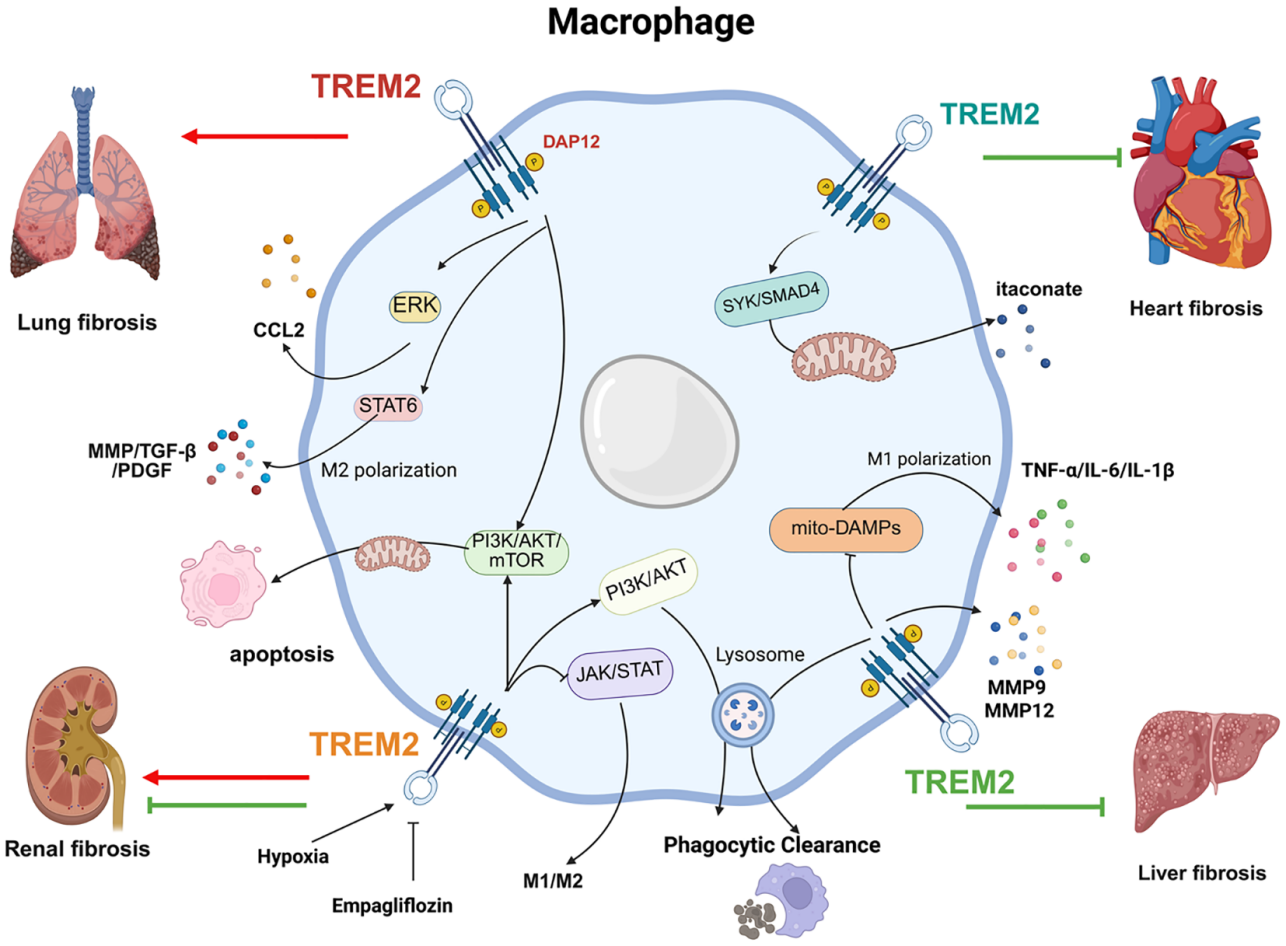
Role of TREM2 expression on macrophages in fibrosis. This figure depicts the generalized signaling framework in which TREM2, via its adaptor protein DAP12, activates universal signaling modules, including the PI3K/AKT/mTOR, JAK/STAT, ERK/STAT6, and SYK/SMAD4 pathways. Such activation modulates conserved macrophage functions—phagocytic clearance, metabolic reprogramming, polarization and inflammatory modulation, cell survival and proliferation, and the secretory profile. These processes lead to altered secretion of proinflammatory cytokines (such as CCL2, TNF-α, IL-6, and IL-1β), matrix metalloproteinases (such as MMP9 and MMP12), and profibrotic factors (such as TGF-β and PDGF). Although these molecular modules are ubiquitous across tissues, their activation in specific pathological microenvironments results in distinct organ-specific outcomes: promoting pulmonary fibrosis, inhibiting cardiac and hepatic fibrosis, and exhibiting dual roles in renal fibrosis. The figure was produced using biorender (https://app.biorender.com). TREM2, triggering receptor expressed on myeloid cells 2; DAP12, DNAX-activating protein 12; SYK, spleen tyrosine kinase; AKT, protein kinase B; ERK, extracellular signal-regulated kinase; mTOR, mechanistic target of rapamycin; TGF-β, transforming growth factor; STAT6, signal transducer and activator of transcription 6; PDGF, platelet-derived growth factor; PI3K, phosphoinositide 3-kinase; JAK, Janus kinase; mito-DAMPs, mitochondrial damage-associated molecular patterns; MMP, matrix metalloproteinase.

The role of TREM2 in fibrosis across various organs demonstrates significant context specificity. This specificity arises from the unique injury patterns, microenvironmental signals, and cellular interaction networks inherent to each organ, which serve as distinct “instructions” that recruit and integrate the underlying functional modules of TREM2. Consequently, this orchestrates pathological outcomes that are either profibrotic or protective/resolving (**Table 1**, **Figure 2**).

**Table 1:** Overview of TREM2 in fibrotic diseases.

Organ	Species	Model/samples	Expression by	Change	Function mechanism	Publication year & Ref.
Lung	Rat	BLM-induced fibrosis	NA	↑	NA	2010 (105)
	Human Mouse	IPF BLM-induced fibrosis	Ams Mo-AMs	↑	Profibrotic: SM-TREM2 activates AKT/ERK/mTOR signaling to promote AM survival and pro-fibrotic mediator secretion (TGF-β1, SPP1, PDGFA, MMP12); TREM2^+^ Mo-AMs inhibit ATII regeneration and differentiation into ATI. Exogenous sTREM2 competitively binds SM, inducing AM apoptosis and alleviating bleomycin-induced pulmonary fibrosis; TREM2 blocking antibody blocks SM’s pro-fibrotic effect and attenuates fibrosis.	2025 (41)
	Human Mouse	IPF BLM-induced fibrosis	M2	↑	Profibrotic: TREM2 silencing blocked STAT6 activation and inhibited M2 macrophage polarization and reduced the level of TGF-β, Fizz, PDGF, Fib, Col I, α-SMA.	2023 (16)
	Human Mouse	IPF BLM-induced fibrosis	MoMs	↑	Profibrotic: TREM2 macrophages may trigger pulmonary fibrosis by sensing dysregulated SM metabolism and promoting cell chemotaxis cell chemotaxis; TREM2 blockade ameliorates fibrosis	2025 (53)
	Human Mouse	IPF SP-C mutation	macrophages	↑	Profibrotic: TREM2^+^ macrophages coordinate pro-fibrotic communication.	2024 (74)
	Mouse	BLM-induced fibrosis	macrophages	↑	Profibrotic: UCMSCs reduce the expression of the gene TREM2.	2024 (75)
	Mouse	BLM-induced fibrosis	macrophages	↓	Anti-fibrotic: Intratracheal injection ADSCs increase TREM2^+^ anti-inflammatory macrophages.	2022 (76)
Liver	Human Mouse	Cirrhotic patients. CCl_4_ models	SAMs	↑	pro-fibrotic: TREM2^+^CD9^+^ SAMs derive from monocytes, driving fibrosis.	2019 (77)
	Mouse	HFC-NAFLD models	MoMs	↑	Anti-fibrotic: TREM2^+^ macrophages localize to inflamed areas, suppress inflammation.	2022 (68)
	Human Mouse	APAP injury models CCl_4_ models	LAMs LAM-like KCs	↑	Anti-fibrotic: TREM2 deficiency impairs dead cell clearance, exacerbating fibrosis.	2025 (54)
	Mouse	Foz+WD models CCl_4_ models	LAMs	↑	Anti-fibrotic: TREM2^+^ macrophages dominate regression; enhance phagocytosis/lipid handling/collagen degradation.	2024 (14)
	Mouse	CCl_4_ induces hepatic fibrosis	macrophages	↑	Anti-fibrotic: TREM2 KO reduces phagocytosis, increases mito-DAMPs promoting M1 polarization, driving fibrosis.	2024 (15)
Heart	Human	CHD patients	NA	↑	NA: Elevated sTREM2 in serum is associated with CV risk factors (TG, HDL-C, ApoB, smoking); sTREM2 may serve as a potential biomarker for CHD.	2023 (81)
	Human Mouse	MI patients MI mice model,	macrophages	↑	Anti-fibrotic: TREM2/SYK/SMAD4 increased itaconate production by decreasing SLC25A53 thereby inhibiting cardiomyocyte apoptosis and promoted fibroblast proliferation. Injecting TREM2 adenovirus could promote MI remodeling.	2024 (61)
	Human Mouse	CAD patients MI mice model	platelet	↓	Anti-fibrotic: ER stress downregulated TREM2 via the CHOP-C/EBPα axis. TREM2 activating antibody (S1P) activated TREM2/DAP12/SHIP1 axis, which inhibits platelet activation by inhibiting PIP3/Akt, thereby reduced thrombosis, and alleviated experimental myocardial infarction.	2025 (79)
	Mouse	MI mice model	macrophages	↑	NA: TREM2 upregulated in macrophages during the late phase post-MI, exerting anti-inflammatory functions. sTREM2 injection improves cardiac structure/function.	2023 (78)
	Human Mouse	CAD patients MI mice model	cardiomyocytes	↑	Anti-fibrotic: TREM2 may activate PI3K/AKT curb myocardial ischemia injury. TREM2 overexpression alleviated cardiac tissue damage in IM mice. Plasma sTREM2 as potential CAD severity/diagnostic biomarker.	2022 (80)
Skin	Human Mouse	SSc patients BLM-induced fibrosis	macrophages	↑	Anti-fibrotic: Genetic ablation of TREM2 in mice globally accelerates and aggravates skin fibrosis, whereas transferring TREM2^hi^ macrophages improves and alleviates skin fibrosis. The disease-associated TREM2^+^ macrophages in skin fibrosis exhibit overlapping signatures with fetal skin counterparts in mice and human to maintain skin homeostasis.	2024 (56)
	Human Mouse	Hypertrophic scarring	CAR-TREM2 macrophages	NA	Anti-fibrotic: CAR-TREM2-macrophages delivered by mMNs targeted DPP4 ^+^ fibroblasts to phagocytose DPP4^+^ fibroblasts and suppress TGF-β secretion and then modulated ECs subtype by suppressing Lrg1^-^ to prevent scarring.	2024 (82)
Kidney	Human	CKD patients	NA	↑	Anti-fibrotic: TREM-1/TREM-2 ratio negatively correlates with fibrosis (Cutoff:1.338); In human moderate-severe fibrosis kidney tissue, the protein expression of TREM1 was lower and the TREM2 was higher than none-mild fibrosis kidney tissue (where “none-mild” was defined as <25% of the renal interstitium).	2021(83)
	Mouse	UUO	BMDM	↑	Anti-fibrotic: TREM2 promotes macrophage apoptosis, augments M1/M2 polarization via JAK-STAT pathway (TGF-β1-dependent), impairs mTOR-mediated survival to achieved anti-fibrotic effects in renal injury; TREM2 deficiency exacerbates fibrosis.	2024(42)
	Mouse	UIRI-AKI-CKD model	macrophages	↑	Anti-fibrotic: TREM2 deficiency exacerbates renal inflammation, injury, and fibrosis in UIRI mice; Hypoxia upregulates TREM2 via HIF-1α to enhance macrophage phagocytosis and reduce pro-inflammatory cytokines and alleviate tubular apoptosis/fibrosis through PI3K-AKT.	2025(66)
	Rats	5/6 nephrectomy	M2 macrophages	↑	Profibrotic: Empagliflozin remarkably inhibited the expression of fibrosis-promoting (IFG1 and TREM2) in CD206CD68 M2 macrophages.	2022(84)
	Human Mouse	Renal fibrosis patients UUO	macrophages	↑	Profibrotic: TREM-2^−/−^ macrophages increase the MMP-9/TIMP-1 ratio in their exosomes via HSPa1b/AKT pathway, degrading ECM and alleviating renal fibrosis; The polyclonal antibodies against TREM-2 effectively relieved UUO-induced renal fibrosis	2024(85)

TREM2, triggering receptor expressed on myeloid cells 2; sTREM2, soluble TREM2; ECM, extracellular matrix; DAP12, DNAX-activating protein 12; SYK, spleen tyrosine kinase; BLM, bleomycin; IPF, idiopathic pulmonary fibrosis; Mo-AM, monocyte-derived alveolar macrophages; SM, sphingomyelin; AKT, protein kinase B; TGF-β1, transforming growth factor β1; SPP1, secreted phosphoprotein 1; APDGFA, platelet-derived growth factor subunit A; MMP12, matrix metalloproteinase 12; ATII, alveolar type II epithelial cells; ATI, alveolar type I epithelial cells; STAT6, signal transducer and activator of transcription 6; CCL2, C-C motif chemokine ligand 2; PDGF, platelet-derived growth factor; Fib, fibronectin; Col I, collagen type I; α-SMA, α-smooth muscle actin; MoMs, monocyte-derived macrophages; UCMSCs, umbilical cord mesenchymal stem cells; ADSCs, adipose-derived stem cells; SP-C, surfactant protein C; HSCs, hepatic stellate cells; NAFLD, nonalcoholic fatty liver disease; LAM, lipid-susceptible.

### 5.1 Lung fibrosis

In pulmonary fibrosis, TREM2 signaling primarily yields a pro-fibrotic net result, particularly during the active development and progressive stages of the disease. This pathogenic influence is facilitated by macrophages, which orchestrate multiple core functional modules, in response to microenvironmental cues. A critical characteristic of the pulmonary fibrosis microenvironment is the disruption of lipid metabolism, notably the aberrant accumulation of sphingomyelin. As a lipid sensor, TREM2 can recognize and bind to sphingomyelin within this microenvironment, thereby activating its downstream AKT/ERK/mTOR signaling pathways (41). This activation promotes cellular survival and proliferation, thereby providing a cellular foundation for the prolonged residence and continuous expansion of monocyte-derived alveolar macrophages (Mo-AMs) within damaged lung tissue (16, 41, 74). As the pathological environment persists, TREM2 further facilitates the polarization of macrophages towards the pro-fibrotic M2 phenotype via the STAT6 pathway (16). The activated TREM2+ macrophages engage in secretory activities, releasing factors such as TGF-β1, SPP1, and PDGFA, which impede epithelial cell repair and ultimately result in irreversible matrix deposition (41). These mechanisms are further substantiated by interventional evidence: knockout or inhibition of the TREM2 gene disrupts macrophage survival and M2 polarization, leading to a reduction in fibrosis markers (16, 53). Furthermore, employing exogenous sTREM2 or neutralizing antibodies to inhibit the interaction between sphingomyelin and TREM2, as well as blocking downstream signal activation, can effectively mitigate fibrosis (41). Mesenchymal stem cells may attenuate pulmonary fibrosis by down-regulating TREM2 expression (75) or by enhancing the anti-inflammatory macrophage subset (76).

### 5.2 Liver fibrosis

In liver fibrosis, the role of TREM2 is characterized by significant stage-dependent variations. During the resolution phase of fibrosis, TREM2 demonstrates a pronounced anti-fibrotic effect. At this juncture, apoptotic liver cells and lipid droplets generated by the damaged liver constitute crucial microenvironmental signals. TREM2, upon activation by these lipid signals, initiates its phagocytic clearance function, effectively engulfing apoptotic liver cells and lipid droplets. The absence of TREM2 results in the accumulation of cellular debris and the release of damage-associated molecular patterns (DAMPs), thereby exacerbating inflammation (15, 54). Concurrently, TREM2^+^ macrophages secrete substantial quantities of matrix-degrading enzymes, including MMP9 and MMP12, which facilitate the degradation of collagen deposits and promote scar resolution (14, 68). In models of metabolic liver disease, TREM2^+^ macrophages localize within inflammatory regions and mitigate the inflammatory response (68). However, during the early stages of disease or in cirrhosis, monocyte-derived TREM2^+^CD9^+^ scar-associated macrophages (SAMs) may adopt a pro-fibrotic phenotype, thereby contributing to disease progression (77).

### 5.3 Cardiac and skin fibrosis

In cardiac and skin fibrosis, TREM2 signaling primarily yields an anti-fibrotic net result. In post-myocardial infarction repair, there is an upregulation of TREM2 expression in macrophages. This upregulation facilitates metabolic reprogramming, leading to increased itaconate production, which in turn supports reparative fibrosis and enhances ventricular remodeling (41, 61). The administration of sTREM2 has been shown to further enhance cardiac function (78). Moreover, TREM2 signaling in platelets plays a role in modulating the thrombo-inflammatory network. ER stress downregulated TREM2 via the CHOP-C/EBPα axis in platelets. Activation of TREM2 with a specific antibody can inhibit platelet activation via the DAP12/SHIP1 axis, thereby mitigating secondary myocardial fibrosis (79). Clinical studies indicate that plasma levels of sTREM2 in patients with coronary heart disease positively correlate with the severity of coronary artery stenosis, highlighting its potential utility as a biomarker (80, 81). In the context of skin fibrosis, TREM2-positive macrophages display transcriptional profiles like those of fetal skin macrophages and exhibit anti-fibrotic properties. A global deficiency in these macrophages accelerates fibrosis, whereas their adoptive transfer ameliorates the condition (56). Building on this concept, engineered CAR-TREM2 macrophage therapy has been developed to specifically target and eliminate DPP4-positive fibroblasts, offering a novel therapeutic approach (82).

### 5.4 Renal fibrosis

Renal models elucidate the complex and dynamic bidirectional nature of TREM2 function. During the acute injury phase, such as unilateral ureteral obstruction (UUO) or ischemia-reperfusion, hypoxia induces TREM2 expression via hypoxia-inducible factor 1-alpha (HIF-1α), which activates its anti-fibrotic functions. These functions include enhancing macrophage phagocytic clearance capacity and reducing inflammation and tubular apoptosis through the PI3K-AKT pathway, while inhibiting the JAK/STAT pathway, thereby mitigating fibrosis (42, 66). Clinical observations have also demonstrated that the urinary TREM-1/TREM-2 ratio in patients with chronic kidney disease is inversely correlated with the degree of tubulointerstitial fibrosis (83). However, in the context of chronic persistent injury, TREM2 may contribute to pro-fibrotic processes, as evidenced by its aberrant expression in M2 macrophages in a 5/6 nephrectomy model (84). Interestingly, in such chronic models, TREM2 deficiency or antibody blockade may promote matrix degradation by modulating macrophage exosomes, such as altering the matrix metalloproteinase-9/tissue inhibitor of metalloproteinases-1 (MMP-9/TIMP-1) ratio, thereby unexpectedly alleviating fibrosis (85).

In conclusion, the involvement of TREM2 in organ fibrosis arises from the differential integration of its universal core functional modules in response to distinct microenvironmental signals. In lung fibrosis, these modules synergistically enhance matrix deposition. Conversely, during the resolution phase of liver fibrosis, they collaborate to initiate repair programs. In cardiac and dermal tissues, they direct reparative remodeling, while in the kidney, their functional output demonstrates distinct stage-dependent dynamic variations. Comprehending this relationship of “universal mechanisms – specific integration” is essential for the development of precise, targeted therapeutic strategies.

## 6 Clinical translations of TREM2 targeting: from biomarkers to therapy

### 6.1 TREM2 and its soluble form as biomarkers of disease activity and prognosis

Because membrane-bound TREM2 is difficult to detect directly and stably in peripheral biofluids, current clinical studies of TREM2 as a biomarker have primarily focused on its soluble form, sTREM2. The clinical utility of sTREM2 as a multifaceted biomarker reflecting the activation status of myeloid cells—such as microglia and macrophages—has demonstrated significant potential across a broad spectrum of inflammatory and degenerative pathologies. Although its precise physiological role remains a subject of ongoing debate—specifically whether it functions as a decoy receptor to competitively inhibit ligand binding or acts as a bioactive ligand to directly mediate signal transduction—fluctuations in sTREM2 levels across biological fluids provide a sensitive readout of changes within the tissue immune microenvironment (**Table 2**).

**Table 2:** Clinical significance and alterations of sTREM2 across various pathologies.

Disease	Sample source	sTREM2 trend	Clinical & pathological implications	Refs
AD	CSF/Plasma	↑	Reflects microglial activation; correlates with *Aβ*/*τ* pathology and cognitive decline.	(86, 87)
PD	CSF	↑	Potential immune-related biomarker for neuronal injury.	(88)
ALS	CSF	↑	Positively correlates with motor neuron damage and disease progression rate.	(89)
MSA	CSF	↑	Closely associated with neuroinflammatory markers (e.g., NfL, GPNMB).	(90)
PACNS	CSF/Serum	↑	Predicts prognosis; reflects the severity of neurological injury.	(91)
Coronary Atherosclerosis	Plasma	↑	Reflects intra-plaque macrophage activity; indicates plaque instability or rupture.	(92)
Cardiovascular Disease	Plasma	↑	Correlates with coronary lesion severity and systemic inflammation.	(72)
MASLD/MASH	Plasma	↑	Biomarker for tracking the progression from simple steatosis to MASH.	(106)
Hepatic fibrosis	Plasma	↑	Predicts advanced fibrosis and post-hepatectomy liver failure (PHLF); linked to poor survival.	(93)
Renal fibrosis	Urine	Ratio ↑*	Increased urinary TREM-1/2 ratio predicts tubulointerstitial fibrosis severity.	(75)
Pediatric IgA Nephropathy	Plasma	↑	Elevated levels significantly correlate with the severity of proteinuria.	(107)

↑, increased levels; Ratio ↑*, specifically refers to the urinary TREM-1 to TREM-2 ratio. AD, Alzheimer’s Disease; PD, Parkinson’s Disease; ALS, Amyotrophic Lateral Sclerosis; MSA, Multiple System Atrophy; PACNS, Primary Angiitis of the Central Nervous System; CHD, Coronary Heart Disease; CAD, Coronary Artery Disease; IgAN, IgA Nephropathy; MASLD, metabolic dysfunction-associated steatotic liver disease; MASH, metabolic dysfunction-associated steatohepatitis; CSF, cerebrospinal fluid; NfL, neurofilament light chain; GPNMB, glycoprotein nonmetastatic melanoma protein B; PHLF, post-hepatectomy liver failure.

In central nervous system pathologies, cerebrospinal fluid (CSF) levels of sTREM2 serve as a crucial biomarker for microglial activation and neurodegeneration. In Alzheimer’s disease (AD), increased sTREM2 levels are closely linked to early Aβ deposition, pathological tau modifications, and subsequent cognitive decline (86, 87). In Parkinson’s disease (PD), it is considered an immune-related marker of neuronal injury (88), while in amyotrophic lateral sclerosis (ALS), its levels show a positive correlation with the extent of motor neuron damage and the rate of disease progression (89). Additionally, in patients with multiple system atrophy (MSA), sTREM2 levels are strongly associated with neuroinflammatory markers, such as neurofilament light chain (NfL) and glycoprotein nonmetastatic melanoma protein B (GPNMB) (90). Beyond chronic conditions, sTREM2 levels in the serum or CSF of patients with primary angiitis of the CNS (PACNS) effectively reflect the severity of neurological injury and serve as a predictor of clinical prognosis (91). Recent research underscores the role of sTREM2 as a sensitive biomarker for assessing systemic myeloid activation, with a particular emphasis on macrophage activity. In individuals diagnosed with coronary heart disease (CHD), there is a significant correlation between circulating sTREM2 levels and both systemic inflammatory scores and the anatomical severity of coronary lesions (72). Additional studies suggest that plasma sTREM2 is indicative of macrophage activity within atherosclerotic plaques, thus serving as a potential marker for assessing plaque instability or the risk of rupture (92).

The potential of sTREM2 as a biomarker extends to metabolic and fibrotic disorders affecting various organs. Notably, sTREM2 levels demonstrate significant utility in monitoring the progression from metabolic dysfunction-associated steatotic liver disease (MASLD) to metabolic dysfunction-associated steatohepatitis (MASH) (81). In individuals with hepatic fibrosis, plasma sTREM2 serves as a predictor of advanced fibrosis severity and is closely associated with the occurrence of post-hepatectomy liver failure (PHLF) and diminished long-term survival (93). In renal diseases, elevated plasma sTREM2 levels in pediatric IgA nephropathy (IgAN) correlate with increased proteinuria severity (93).

Furthermore, ratio-based indicators and combined biomarker panels related to TREM2 provide greater specificity for evaluating disease activity and prognosis. The urinary TREM-1/sTREM2 ratio has been identified as a reliable predictor of renal tubulointerstitial fibrosis severity (75). In lung tissue from patients with chronic obstructive pulmonary disease (COPD), the TREM2/TREM1 mRNA ratio is increased and correlates with disease severity, including a decline in FEV_1_, and then this ratio is significantly associated with increased CHIT1 mRNA levels, suggesting that CHIT1 may serve as an auxiliary marker of pulmonary TREM2-related inflammatory responses (94). In addition, after treatment with a TREM2 agonist such as hPara.09, increased CSF CHI3L1 levels accompanied by decreased sTREM2 were observed, together with significant transient microglial proliferation and clustering. These findings suggest that the combined measurement of CSF CHI3L1 and sTREM2 may more specifically reflect the response of microglia to TREM2 activation and could be useful for monitoring target engagement in clinical trials (95).

While TREM2-related biomarkers have shown significant relevance and potential across various diseases, their development as precise diagnostic and stratification tools remains challenged by several factors. Firstly, the sources of sTREM2 are heterogeneous, as it can be produced through diverse mechanisms, such as ADAM family-mediated ectodomain shedding and alternative splicing, each potentially having distinct biological implications. Future research should incorporate isoform-specific detection techniques and integrate analyses of tissue origin with downstream signaling activation profiles to enhance the accuracy of sTREM2 as a disease-specific predictive biomarker. Secondly, the diagnostic efficacy of sTREM2 in isolation may be limited. More robust assessments could be achieved by measuring the TREM2/TREM1 ratio or employing multimarker panels that combine sTREM2 with CHI3L1, CHIT1, and other indicators, thereby facilitating a more comprehensive evaluation of inflammatory status, tissue injury, and disease progression.

### 6.2 TREM2-targeted antifibrotic therapy: preclinical strategies and translational potential

Given the complex role of TREM2 in organ fibrosis, therapeutic strategies should aim to “precise correction of specific pathological states”. This leads to two complementary preclinical research directions: inhibiting TREM2 in environments where it promotes fibrosis and enhancing its function when its protective effects are lacking (**Table 3**).

**Table 3:** Overview of preclinical research on TREM2-targeted drugs in organ fibrosis.

Drug/intervention	Disease model	Mechanism	Ref.
Inhibitory strategies			
Anti-TREM2 antibody	BLM induced-lung fibrosis	Competitively inhibits sphingomyelin binding	(41)
	UUO-induced renal fibrosis	Targets the TREM2-HSPa1b/ACT/Shaft pathway to mitigate ECM remodeling.	(85)
Exogenous sTREM2	BLM induced-lung fibrosis	Competitively inhibits membrane receptor signaling via tracheal delivery.	(41)
Empagliflozin	5/6 Nephrectomy model	Inhibited the expression of TREM2	(84)
UCMSCs	BLM induced-lung fibrosis	Downregulates TREM2 expression in macrophages	(75)
Agonistic strategies			
ADSCs	BLM induced-lung fibrosis	Increase TREM2^+^ anti-inflammatory macrophages	(76)
Infusion of TREM2-positive macrophages	UIRI-AKI-CKD model	Enhances phagocytic clearance	(66)
	BLM induced-skin fibrosis	–	(56)
CAR-TREM2 macrophages	Scarring model	Targets and removes DPP4-positive fibroblasts by microneedling delivery	(82)
TREM2 adenovirus	MI mice model	Activates the TREM2/SYK/SMAD4/itaconate pathway to inhibit cardiomyocyte apoptosis and promoted fibroblast proliferation	(61)
S1P (TREM2 activating antibody)	MI mice model	Activates the DAP12/SHIP1 axis to inhibit platelet activation, blocking the thrombosis-inflammation-fibrosis cascade	(79)
Exogenous sTREM2	MI mice model	sTREM2 injection improves cardiac structure/function	(78)

TREM2, triggering receptor expressed on myeloid cells 2; ADSCS, adipose-derived mesenchymal stem cells; AKI, acute kidney injury; BLM, bleomycin; CAR, chimeric antigen receptor; CKD, chronic kidney disease; DPP4, dipeptidyl peptidase-4; ECM, extracellular matrix; MI, myocardial infarction; sTREM2, soluble TREM2; S1P, sphingosine-1-phosphate; SYK, spleen-associated tyrosine kinase; UCMSCS, umbilical cord mesenchymal stem cells; UIRI, unilateral ischemia–reperfusion injury; UUO, unilateral ureteral obstruction.

#### 6.2.1 Inhibitory strategies

When TREM2 exhibits profibrotic properties in specific organs (e.g., lungs and chronic kidney disease stages), inhibiting its activity becomes a rational strategy.

##### 6.2.1.1 Antibody blockade

In pulmonary fibrosis models, neutralizing antibodies that target TREM2 can competitively inhibit its interaction with the ligand sphingomyelin. This effectively reduces the profibrotic activation of macrophages and collagen deposition, thereby demonstrating direct therapeutic potential (41). In chronic renal fibrosis, anti-TREM2 antibodies have also been shown to modulate the balance of matrix metabolism by disrupting the TREM2-HSPa1b/AKT signaling pathway (85).

##### 6.2.1.2 Soluble receptor intervention

Exogenously administered soluble TREM2 can function as a “decoy receptor,” competitively binding to its ligands, such as sphingomyelin, and thus inhibiting the downstream signaling mediated by membrane-bound TREM2. This strategy has been shown to mitigate fibrosis progression in models of lung fibrosis (41).

##### 6.2.1.3 Small molecule and MSCs interventions

Indirectly regulating receptor expression is a viable approach. For instance, empagliflozin inhibits TREM2 expression, demonstrating antifibrotic effects in a nephrectomy model (84). Additionally, the antifibrotic benefits of umbilical cord-derived MSC therapy are associated with the downregulation of TREM2 expression in macrophages (75).

#### 6.2.2 Enhancing strategies

In contexts where TREM2 function is insufficient or where it plays a protective role (e.g., acute kidney injury, the regression phase of liver fibrosis, and cardiac repair), enhancing its activity is another core strategy.

##### 6.2.2.1 Adoptive cell transplantation and engineering

The adoptive transfer of macrophages with elevated TREM2 expression represents a direct approach to functional supplementation. In models of acute kidney injury, the infusion of TREM2-positive macrophages has been shown to significantly increase the phagocytic clearance capacity and facilitate tissue repair (42, 66). A more sophisticated strategy involves engineered cell therapy. For example, in skin fibrosis, CAR-TREM2 macrophages administered via MNs are designed to specifically recognize and eliminate profibrotic DPP4^+^ fibroblasts, thereby achieving precise remodeling of the pathological microenvironment (82).

##### 6.2.2.2 Agonist application

The development of TREM2 agonists constitutes a significant area of pharmacological research. In myocardial fibrosis models, for example, the agonist S1P effectively inhibited the platelet-mediated “thrombosis–inflammation–fibrosis” cascade through activation of the TREM2/DAP12/SHIP1 axis (79). These findings offer a proof-of-concept for the development of small molecule agonists that can be administered orally or via injection.

### 6.3 Fundamental challenges in clinical translation

Despite preclinical research indicating the significant potential of TREM2 as a therapeutic target for fibrosis, its clinical translation faces substantial challenges. The complexity of these challenges is underscored by the developmental trajectories of leading drugs in other disease domains.

#### 6.3.1 The precision control dilemma arises from “functional bidirectionality”

TREM2 can exert opposing effects at various stages of disease progression within the same organ, necessitating that therapeutic interventions possess spatiotemporal specificity or environmental adaptability. Employing straightforward systemic agonism or inhibition could yield contradictory or detrimental outcomes at different stages of the disease. The foremost scientific challenge lies in the development of pharmacological agents capable of “intelligently” discerning the pathological microenvironment and responding in a context-appropriate manner.

#### 6.3.2 The antagonistic relationship between TREM1 and TREM2

TREM1 predominantly functions to amplify acute inflammatory responses, whereas TREM2 is more closely associated with the regulation of chronic inflammation and the maintenance of immune homeostasis. Although both receptors may be modulated by TLR4-related signaling pathways in specific contexts, they produce opposing downstream biological effects (96). Consequently, the dynamic equilibrium between TREM1 and TREM2 may collectively influence the magnitude of local inflammatory responses and the outcomes of tissue repair. In the context of cardiovascular disease, the balance between TREM1 and TREM2 has been significantly correlated with the inflammatory burden and the severity of tissue injury; both the inhibition of TREM1 (e.g., via the LR12 peptide) and the activation of TREM2 (e.g., through AL002 and VG-3927) have demonstrated therapeutic potential in preclinical models (97). The key translational challenge lies in determining how to modulate this axis in a stage-specific and context-dependent manner without disrupting immune homeostasis.

#### 6.3.3 Systemic network complexity and functional redundancy

From a systems biology and philosophical standpoint in therapeutic design, TREM2 does not function in isolation. In the critical process of recognizing and clearing apoptotic cells and cellular debris (efferocytosis), TREM2 demonstrates significant functional redundancy with other phagocytic receptors, particularly MerTK, a notable member of the TAM receptor family. This intrinsic biological redundancy poses substantial challenges to the pharmacodynamics of therapies targeting TREM2 alone. Pharmacological activation or inhibition of TREM2 may induce compensatory expression or adaptation of parallel receptors such as MerTK, thereby reducing the expected therapeutic efficacy. Furthermore, disrupting this intricate, multi-receptor-controlled homeostatic network risks initiating unforeseen off-target inflammatory cascades or causing immunological instability. As a result, the effective translation of TREM2-targeted anti-fibrotic therapies requires a conceptual paradigm shift from a reductionist approach focused on single targets to a systems network pharmacology framework. This shift presents an additional significant challenge in addressing the body’s redundant biological safety mechanisms.

#### 6.3.4 Lessons and warnings: challenges in drug development for neurological diseases

In the domain of AD, the clinical advancement of several TREM2-targeted agonists, such as AL-002, DNL-919, and ILUZANEBART, has faced significant challenges, including failure to achieve primary endpoints, discontinuation due to toxicity (e.g., hematotoxicity), and lack of clinical benefit (98–101). These instances underscore the necessity of addressing common obstacles such as drug delivery efficiency, particularly brain penetration, precise regulation of various TREM2 activation states, and unexpected off-target toxicity for successful translation. While recent developments, including VHB-937 and the oral small-molecule agonist VG3927, present new opportunities (102–104), their long-term efficacy and safety must still be confirmed through large-scale clinical trials. The clinical challenges observed in neurological diseases offer a complex reference point for understanding fibrosis. Although the pharmacological obstacles differ markedly due to the “Barrier Quotient”—where the restrictive nature of the blood-brain barrier (BBB) necessitates high, often toxic systemic doses, in contrast to the naturally increased vascular permeability in fibrotic organs such as the liver and lungs—the biological challenge of “Macrophage Heterogeneity” remains a significant common obstacle. Similar to how TREM2-dependent plaque clearance in AD is advantageous in the early stages but potentially inflammatory in later stages, the dynamic roles of TREM2^+^ macrophages in fibrosis indicate that merely improving organ access is inadequate. Without precise timing to exploit the “window-of-opportunity,” systemic TREM2 agonism may paradoxically worsen rather than ameliorate the fibrotic environment.

#### 6.3.5 Organ-targeted delivery and off-target effects

Considering that TREM2 is widely expressed across various myeloid cell populations, including osteoclasts in the bone and specialized macrophages in the spleen, systemic administration presents considerable risks to bone homeostasis and immune surveillance. This necessitates a shift towards organ-specific delivery methods to ensure clinical safety. In addressing liver fibrosis, ligand-modified nanocarriers such as lipid nanoparticles (LNPs) or exosomes functionalized with mannose or dextran can be employed to target CD206 receptors on Kupffer cells. This approach focuses the therapeutic agent within the hepatic environment while minimizing exposure to bone and spleen tissues. Similarly, pulmonary fibrosis can be managed through localized inhalation techniques using dry powder inhalers (DPI) or nebulized nanocapsules, which deliver the treatment directly to alveolar macrophages. This method bypasses systemic circulation, thereby reducing the risk of hematotoxicity observed in Alzheimer’s disease trials. For renal targeting, the use of KIM-1-responsive polymers or size-selective nanoparticles can be optimized. These can be further enhanced with “smart” pH-sensitive or reactive oxygen species (ROS)-responsive linkers, ensuring that the TREM2 agonist is released exclusively within the oxidative and acidic microenvironment of the fibrotic lesion, thereby protecting healthy tissue.

## 7 Conclusion and future perspectives

Research on TREM2 has evolved from initial genetic association studies to a comprehensive understanding of its pivotal role as a central regulator of macrophage function. In essence, TREM2 adheres to a “universal-specificity” paradigm in organ fibrosis, functioning as a universal sensory-effector system that integrates organ- and stage-specific microenvironmental cues—such as sphingomyelin or apoptotic debris—to yield diverse pro- or anti-fibrotic outcomes. To effectively translate these findings from bench to bedside, future research must focus on several critical areas (1): Deciphering dynamic control and signal integration: A significant challenge is to unravel the spatiotemporal regulation of TREM2. It is imperative to elucidate how upstream signals—including specific ligand combinations, metabolic environments, and cell-cell interactions—are integrated to drive the phenotypic transition of TREM2^+^ macrophages between pro-fibrotic and reparative states during the recruitment, activation, and resolution phases. (2) The unresolved role of soluble TREM2: It is imperative to elucidate the precise pathological role of sTREM2, determining whether it functions as an inert byproduct, a competitive decoy receptor, or an independent signaling molecule. A comprehensive understanding of its dynamics in human fibrotic diseases is crucial for establishing its validity as a biomarker and therapeutic target. (3) Promising Translational horizons: ① conditional macrophage therapy: The application of synthetic biology to engineer engineered macrophages that can detect and respond to local fibrotic cues (such as matrix fragments or stress signals) by dynamically modulating TREM2-related reparative pathways. ② precision small molecule and antibody therapeutics: The development of modulators that leverage the structural dynamics of the TREM2 complex to selectively enhance clearance functions or inhibit pro-fibrotic signaling pathways. In conclusion, a thorough comprehension of TREM2-mediated microenvironmental sensing is essential for providing the scientific foundation necessary to develop safe and effective targeted interventions for fibrotic diseases.

## References

[1] Odell ID Steach H Gauld SB Reinke-Breen L Karman J Carr TL . Epiregulin is a dendritic cell-derived EGFR ligand that maintains skin and lung fibrosis. Sci Immunol. (2022) 7:eabq6691. doi: 10.1126/sciimmunol.abq6691 36490328 PMC9840167

[2] Huang E Peng N Xiao F Hu D Wang X Lu L . The roles of immune cells in the pathogenesis of fibrosis. Int J Mol Sci. (2020) 21 (15):5203. doi: 10.3390/ijms21155203 32708044 PMC7432671

[3] Artlett CM . The mechanism and regulation of the NLRP3 inflammasome during fibrosis. Biomolecules. (2022) 12 (5):634. doi: 10.3390/biom12050634 35625564 PMC9138796

[4] Savin IA Zenkova MA Sen'kova AV . Pulmonary fibrosis as a result of acute lung inflammation: Molecular mechanisms, relevant *in vivo* models, prognostic and therapeutic approaches. Int J Mol Sci. (2022) 23 (23):14959. doi: 10.3390/ijms232314959 36499287 PMC9735580

[5] Mack M . Inflammation and fibrosis. Matrix Biology: J Int Soc For Matrix Biol. (2018) 68-69:106–21. doi: 10.1016/j.matbio.2017.11.010 29196207

[6] Koudstaal T Funke-Chambour M Kreuter M Molyneaux PL Wijsenbeek MS . Pulmonary fibrosis: From pathogenesis to clinical decision-making. Trends Mol Med. (2023) 29:1076–87. doi: 10.1016/j.molmed.2023.08.010 37716906

[7] Czubryt MP Hale TM . Cardiac fibrosis: Pathobiology and therapeutic targets. Cell Signalling. (2021) 85:110066. doi: 10.1016/j.cellsig.2021.110066 34146658 PMC8355135

[8] Humphreys BD . Mechanisms of renal fibrosis. Annu Rev Physiol. (2018) 80:309–26. doi: 10.1146/annurev-physiol-022516-034227 29068765

[9] Guo X Zhu Y Sun Y Li X . IL-6 accelerates renal fibrosis after acute kidney injury via DNMT1-dependent FOXO3a methylation and activation of Wnt/β-catenin pathway. Int Immunopharmacol. (2022) 109:108746. doi: 10.1016/j.intimp.2022.108746 35569307

[10] Black LM Lever JM Agarwal A . Renal inflammation and fibrosis: A double-edged sword. J Histochem Cytochemistry: Off J Histochem Soc. (2019) 67:663–81. doi: 10.1369/0022155419852932 31116067 PMC6713973

[11] Hwang M Savarin C Kim J Powers J Towne N Oh H . Trem2 deficiency impairs recovery and phagocytosis and dysregulates myeloid gene expression during virus-induced demyelination. J Neuroinflamm. (2022) 19:267. doi: 10.1186/s12974-022-02629-1 36333761 PMC9635103

[12] Wang S Cao C Peng D . The various roles of TREM2 in cardiovascular disease. Front Immunol. (2025) 16:1462508. doi: 10.3389/fimmu.2025.1462508 40083551 PMC11903262

[13] Fang C Zhong R Lu S Yu G Liu Z Yan C . TREM2 promotes macrophage polarization from M1 to M2 and suppresses osteoarthritis through the NF-κB/CXCL3 axis. Int J Biol Sci. (2024) 20:1992–2007. doi: 10.7150/ijbs.91519 38617547 PMC11008261

[14] Ganguly S Rosenthal SB Ishizuka K Troutman TD Rohm TV Khader N . Lipid-associated macrophages' promotion of fibrosis resolution during MASH regression requires TREM2. PNAS. (2024) 121:e2405746121. doi: 10.1073/pnas.2405746121 39172787 PMC11363294

[15] Shan S Chao S Liu Z Wang S Liu Z Zhang C . TREM2 protects against inflammation by regulating the release of mito-DAMPs from hepatocytes during liver fibrosis. Free Radic Biol Med. (2024) 220:154–65. doi: 10.1016/j.freeradbiomed.2024.05.004 38710340

[16] Luo Q Deng D Li Y Shi H Zhao J Qian Q . TREM2 insufficiency protects against pulmonary fibrosis by inhibiting M2 macrophage polarization. Int Immunopharmacol. (2023) 118:110070. doi: 10.1016/j.intimp.2023.110070 37003186

[17] Li R Wang X He P . The most prevalent rare coding variants of TREM2 conferring risk of Alzheimer's disease: A systematic review and meta-analysis. Exp Ther Med. (2021) 21:347. doi: 10.3892/etm.2021.9778 33732320 PMC7903442

[18] Jonsson T Stefansson H Steinberg S Jonsdottir I Jonsson PV Snaedal J . Variant of TREM2 associated with the risk of Alzheimer's disease. N Engl J Med. (2013) 368:107–16. doi: 10.1056/nejmoa1211103 23150908 PMC3677583

[19] Guerreiro R Wojtas A Bras J Carrasquillo M Rogaeva E Majounie E . TREM2 variants in Alzheimer's disease. N Engl J Med. (2013) 368:117–27. doi: 10.1136/jnnp-2014-308883.7 PMC3631573 23150934

[20] Scheiblich H Eikens F Wischhof L Opitz S Jüngling K Cserép C . Microglia rescue neurons from aggregate-induced neuronal dysfunction and death through tunneling nanotubes. Neuron. (2024) 112:3106–3125.e8. doi: 10.1016/j.neuron.2024.06.029 39059388

[21] Mishra S Swain PS Pati S Dehury B . Extracellular domain of TREM2 possess two distinct ligand recognition sites: Insights from machine-learning guided docking and all-atoms molecular dynamics simulations. Heliyon. (2025) 11:e41414. doi: 10.1016/j.heliyon.2024.e41414 39866401 PMC11759634

[22] Thornton P Sevalle J Deery MJ Fraser G Zhou Y Ståhl S . TREM2 shedding by cleavage at the H157-S158 bond is accelerated for the Alzheimer's disease-associated H157Y variant. EMBO Mol Med. (2017) 9:1366–78. doi: 10.15252/emmm.201707673 28855301 PMC5623839

[23] Feuerbach D Schindler P Barske C Joller S Beng-Louka E Worringer KA . ADAM17 is the main sheddase for the generation of human triggering receptor expressed in myeloid cells (hTREM2) ectodomain and cleaves TREM2 after Histidine 157. Neurosci Lett. (2017) 660:109–14. doi: 10.1016/j.neulet.2017.09.034 28923481

[24] Zhong Z Ulmschneider MB Lorenz CD . Unraveling the molecular dance: Insights into TREM2/DAP12 complex formation in Alzheimer's disease through molecular dynamics simulations. ACS Omega. (2024) 9:28715–25. doi: 10.1021/acsomega.4c03060 38973875 PMC11223195

[25] Liu Y Theil S Ibach M Walter J . DAP12 interacts with RER1 and is retained in the secretory pathway before assembly with TREM2. Cell Mol Life Sciences: CMLS. (2024) 81:302. doi: 10.1007/s00018-024-05298-w 39008111 PMC11335228

[26] Sudom A Talreja S Danao J Bragg E Kegel R Min X . Molecular basis for the loss-of-function effects of the Alzheimer's disease-associated R47H variant of the immune receptor TREM2. J Biol Chem. (2018) 293:12634–46. doi: 10.1074/jbc.ra118.002352 29794134 PMC6093241

[27] Dantas PHS Matos AO Colmenares MTC Costa VAF Felice AG Neto JRC . Protein recognition is chiefly mediated by the CDR2 region in TREM2 - an in silico characterization. J Mol Graphics Modell. (2025) 138:109058. doi: 10.1016/j.jmgm.2025.109058 40280075

[28] Greven JA Wydra JR Greer RA Zhi C Price DA Svoboda JD . Biophysical mapping of TREM2-ligand interactions reveals shared surfaces for engagement of multiple Alzheimer's disease ligands. Mol Neurodegener. (2025) 20:3. doi: 10.1186/s13024-024-00795-9 39789647 PMC11721465

[29] McMillan IO Liang L Su G Song X Drago K Yang H . TREM2 on microglia cell surface binds to and forms functional binary complexes with heparan sulfate modified with 6-O-sulfation and iduronic acid. J Biol Chem. (2024) 300:107691. doi: 10.1016/j.jbc.2024.107691 39159814 PMC11416269

[30] Ibach M Mathews M Linnartz-Gerlach B Theil S Kumar S Feederle R . A reporter cell system for the triggering receptor expressed on myeloid cells 2 reveals differential effects of disease-associated variants on receptor signaling and activation by antibodies against the stalk region. Glia. (2021) 69:1126–39. doi: 10.1002/glia.23953 33314333

[31] Konishi H Kiyama H . Microglial TREM2/DAP12 signaling: A double-edged sword in neural diseases. Front Cell Neurosci. (2018) 12:206. doi: 10.3389/fncel.2018.00206 30127720 PMC6087757

[32] Cui X Qiao J Liu S Wu M Gu W . Mechanism of TREM2/DAP12 complex affecting β-amyloid plaque deposition in Alzheimer's disease modeled mice through mediating inflammatory response. Brain Res Bull. (2021) 166:21–8. doi: 10.1016/j.brainresbull.2020.10.006 33053435

[33] Beckmann N Neuhaus A Zurbruegg S Volkmer P Patino C Joller S . Genetic models of cleavage-reduced and soluble TREM2 reveal distinct effects on myelination and microglia function in the cuprizone model. J Neuroinflamm. (2023) 20:29. doi: 10.1186/s12974-022-02671-z 36755323 PMC9909920

[34] Traub J Beyersdorf N Sell R Frantz S Störk S Stoll G . Plasma levels of sTREM2 in chronic heart failure: Predictors and prognostic relevance. Am J Physiol Heart Circ Physiol. (2025) 328:H594–602. doi: 10.1152/ajpheart.00728.2024 39918245

[35] Bosco DB Kremen V Haruwaka K Zhao S Wang L Ebner BA . Microglial TREM2 promotes phagocytic clearance of damaged neurons after status epilepticus. Brain Behavior Immun. (2025) 123:540–55. doi: 10.1016/j.bbi.2024.09.034 39353548 PMC11924143

[36] Brennan FH Swarts EA Kigerl KA Mifflin KA Guan Z Noble BT . Microglia promote maladaptive plasticity in autonomic circuitry after spinal cord injury in mice. Sci Transl Med. (2024) 16:eadi3259. doi: 10.1126/scitranslmed.adi3259 38865485

[37] Liu L Wang D Li X Adetula AA Khan A Zhang B . Long-lasting effects of lipopolysaccharide on the reproduction and splenic transcriptome of hens and their offspring. Ecotoxicology Environ Saf. (2022) 237:113527. doi: 10.1016/j.ecoenv.2022.113527 35453024

[38] Hall SC Agrawal DK . Increased TREM-2 expression on the subsets of CD11c(+) cells in the lungs and lymph nodes during allergic airway inflammation. Sci Rep. (2017) 7:11853. doi: 10.1038/s41598-017-12330-6 28928485 PMC5605689

[39] Han S Li X Xia N Zhang Y Yu W Li J . Myeloid Trem2 dynamically regulates the induction and resolution of hepatic ischemia-reperfusion injury inflammation. Int J Mol Sci. (2023) 24 (7):6348. doi: 10.3390/ijms24076348 37047321 PMC10094065

[40] Zhu D Huang M Shen P Zhang B Chen G Chen J . TREM2 expression promotes liver and peritoneal M2 macrophage polarization in mice infected with Schistosoma japonicum. J Cell Mol Med. (2023) 27 (15):2261–9. doi: 10.1111/jcmm.17842 PMC10399532 37430471

[41] Cui H Banerjee S Xie N Hussain M Jaiswal A Liu H . TREM2 promotes lung fibrosis via controlling alveolar macrophage survival and pro-fibrotic activity. Nat Commun. (2025) 16:1761. doi: 10.1038/s41467-025-57024-0 39971937 PMC11840137

[42] Cui Y Chen C Tang Z Yuan W Yue K Cui P . TREM2 deficiency aggravates renal injury by promoting macrophage apoptosis and polarization via the JAK-STAT pathway in mice. Cell Death Dis. (2024) 15:401. doi: 10.1038/s41419-024-06756-w 38849370 PMC11161629

[43] Zheng JY Li XX Lin WY Su S Wu HC Hu RD . Huang-Lian-Jie-Du decoction alleviates depressive-like behaviors in dextran sulfate sodium-induced colitis mice via Trem2/Dap12 pathway. J Ethnopharmacol. (2023) 315:116658. doi: 10.1016/j.jep.2023.116658 37263316

[44] Qin Q Teng Z Liu C Li Q Yin Y Tang Y . TREM2, microglia, and Alzheimer's disease. Mech Ageing Dev. (2021) 195:111438. doi: 10.1016/j.mad.2021.111438 33516818

[45] Zhao P Xu Y Fan X Li L Li X Arase H . Discovery and engineering of an anti-TREM2 antibody to promote amyloid plaque clearance by microglia in 5XFAD mice. mAbs. (2022) 14:2107971. doi: 10.1080/19420862.2022.2107971 35921534 PMC9354770

[46] Zhao N Qiao W Li F Ren Y Zheng J Martens YA . Elevating microglia TREM2 reduces amyloid seeding and suppresses disease-associated microglia. J Exp Med. (2022) 219 (12):e20212479. doi: 10.1084/jem.20212479 36107206 PMC9481739

[47] Li C Zhao B Lin C Gong Z An X . TREM2 inhibits inflammatory responses in mouse microglia by suppressing the PI3K/NF-κB signaling. Cell Biol Int. (2019) 43:360–72. doi: 10.1002/cbin.10975 29663649 PMC7379930

[48] Jay TR von Saucken VE Landreth GE . TREM2 in neurodegenerative diseases. Mol Neurodegener. (2017) 12:56. doi: 10.1186/s13024-017-0197-5 28768545 PMC5541421

[49] Jaitin DA Adlung L Thaiss CA Weiner A Li B Descamps H . Lipid-associated macrophages control metabolic homeostasis in a Trem2-dependent manner. Cell. (2019) 178:686–698.e14. doi: 10.1016/j.cell.2019.05.054 31257031 PMC7068689

[50] Wei W Qu ZL Lei L Zhang P . TREM2-mediated macrophage glycolysis promotes skin wound angiogenesis via the Akt/mTOR/HIF-1α signaling axis. Curr Med Sci. (2024) 44:1280–92. doi: 10.1007/s11596-024-2946-3 39672999

[51] Di Luccia B Molgora M Khantakova D Jaeger N Chang HW Czepielewski RS . TREM2 deficiency reprograms intestinal macrophages and microbiota to enhance anti-PD-1 tumor immunotherapy. Sci Immunol. (2024) 9:eadi5374. doi: 10.1126/sciimmunol.adi5374 38758808 PMC11299520

[52] Hou J Zhang J Cui P Zhou Y Liu C Wu X . TREM2 sustains macrophage-hepatocyte metabolic coordination in nonalcoholic fatty liver disease and sepsis. J Clin Invest. (2021) 131. doi: 10.1172/jci135197 33586673 PMC7880419

[53] Gu X Kang H Cao S Tong Z Song N . Blockade of TREM2 ameliorates pulmonary inflammation and fibrosis by modulating sphingolipid metabolism. Trans Research: J Lab Clin Med. (2025) 275:1–17. doi: 10.1016/j.trsl.2024.10.002 39490681

[54] De Ponti FF Bujko A Liu Z Collins PJ Schuermans S Maueroder C . Spatially restricted and ontogenically distinct hepatic macrophages are required for tissue repair. Immunity. (2025) 58:362–380.e10. doi: 10.1016/j.immuni.2025.01.002 39862865

[55] Medd M . TREM2 in regulating macrophage inflammatory responses and disease pathogenesis. Crit Rev Immunol. (2025) 45:15–24. doi: 10.1615/critrevimmunol.2024054889 39976515

[56] Liang Y Hu Y Zhang J Song H Zhang X Chen Y . Dynamic pathological analysis reveals a protective role against skin fibrosis for TREM2-dependent macrophages. Theranostics. (2024) 14:2232–45. doi: 10.2139/ssrn.4687138 PMC10945340 38505612

[57] Lin CC Chang TY Lu YC Wu YS Huang W Lo WC . TREM-2 mediates dendritic cell-induced NO to suppress Th17 activation and ameliorate chronic kidney diseases. J Mol Med (Berlin Germany). (2022) 100:917–31. doi: 10.1007/s00109-022-02201-7 35532794

[58] Weng Y Wang H Li L Feng Y Xu S Wang Z . Trem2 mediated Syk-dependent ROS amplification is essential for osteoclastogenesis in periodontitis microenvironment. Redox Biol. (2021) 40:101849. doi: 10.1016/j.redox.2020.101849 33486152 PMC7823053

[59] Patterson MT Xu Y Hillman H Osinski V Schrank PR Kennedy AE . Trem2 agonist reprograms foamy macrophages to promote atherosclerotic plaque stability-brief report. Arteriosclerosis Thrombosis Vasc Biol. (2024) 44:1646–57. doi: 10.1161/atvbaha.124.320797 38695172 PMC11208052

[60] Guo X Li B Wen C Zhang F Xiang X Nie L . TREM2 promotes cholesterol uptake and foam cell formation in atherosclerosis. Cell Mol Life Sciences: CMLS. (2023) 80:137. doi: 10.1007/s00018-023-04786-9 37133566 PMC11071710

[61] Gong S Zhai M Shi J Yu G Lei Z Shi Y . TREM2 macrophage promotes cardiac repair in myocardial infarction by reprogramming metabolism via SLC25A53. Cell Death Differ. (2024) 31:239–53. doi: 10.1038/s41418-023-01252-8 38182899 PMC10850484

[62] Riquelme SA Prince A . Airway immunometabolites fuel Pseudomonas aeruginosa infection. Respir Res. (2020) 21:326. doi: 10.1186/s12931-020-01591-x 33302964 PMC7731785

[63] Henderson J Duffy L Stratton R Ford D O'Reilly S . Metabolic reprogramming of glycolysis and glutamine metabolism are key events in myofibroblast transition in systemic sclerosis pathogenesis. J Cell Mol Med. (2020) 24:14026–38. doi: 10.1111/jcmm.16013 33140521 PMC7754020

[64] Lampropoulou V Sergushichev A Bambouskova M Nair S Vincent EE Loginicheva E . Itaconate links inhibition of succinate dehydrogenase with macrophage metabolic remodeling and regulation of inflammation. Cell Metab. (2016) 24:158–66. doi: 10.1016/j.cmet.2016.06.004 27374498 PMC5108454

[65] Zhang X Chen X Zhang L Sun Y Liang Y Li H . Role of trigger receptor 2 expressed on myeloid cells in neuroinflammation-neglected multidimensional regulation of microglia. Neurochem Int. (2023) 171:105639. doi: 10.1016/j.neuint.2023.105639 37926352

[66] Zhang Y Liu Y Luo S Liang H Guo C Du Y . An adoptive cell therapy with TREM2-overexpressing macrophages mitigates the transition from acute kidney injury to chronic kidney disease. Clin Transl Med. (2025) 15:e70252. doi: 10.1002/ctm2.70252 40000418 PMC11859120

[67] Awuah WA Ben-Jaafar A Kong JSH Sanker V Shah MH Poornaselvan J . Novel insights into the role of TREM2 in cerebrovascular diseases. Brain Res. (2025) 1846:149245. doi: 10.1016/j.brainres.2024.149245 39305972

[68] Hendrikx T Porsch F Kiss MG Rajcic D Papac-Miličević N Hoebinger C . Soluble TREM2 levels reflect the recruitment and expansion of TREM2(+) macrophages that localize to fibrotic areas and limit NASH. J Hepatol. (2022) 77:1373–85. doi: 10.1016/j.jhep.2022.06.004 35750138

[69] Chen Y Zeng Z Wei Z Zhan Y Wu L Zhu X . Macrophage Trem2 deficiency aggravates aging-induced vascular remodeling by acting as a non-classical receptor of interleukin-13. Mol BioMed. (2025) 6:153. doi: 10.1186/s43556-025-00377-1 41460571 PMC12748396

[70] Clark D Brazina S Miclau T Park S Hsieh CL Nakamura M . Age-related changes to macrophage subpopulations and TREM2 dysregulation characterize attenuated fracture healing in old mice. Aging Cell. (2024) 23:e14212. doi: 10.1111/acel.14212 38825965 PMC11488338

[71] Rachmian N Medina S Cherqui U Akiva H Deitch D Edilbi D . Identification of senescent, TREM2-expressing microglia in aging and Alzheimer's disease model mouse brain. Nat Neurosci. (2024) 27:1116–24. doi: 10.1038/s41593-024-01620-8 38637622

[72] Tran KM Kawauchi S Kramár EA Rezaie N Liang HY Sakr JS . A Trem2(R47H) mouse model without cryptic splicing drives age- and disease-dependent tissue damage and synaptic loss in response to plaques. Mol Neurodegener. (2023) 18:12. doi: 10.1186/s13024-023-00598-4 36803190 PMC9938579

[73] Wang Z Zhang Y Li X Xia N Han S Pu L . Targeting myeloid Trem2 reprograms the immunosuppressive niche and potentiates checkpoint immunotherapy in NASH-driven hepatocarcinogenesis. Cancer Immunol Res. (2025) 13:1516–32. doi: 10.1158/2326-6066.cir-24-1088 40748990

[74] Moos PJ Cheminant JR Cowman S Noll J Wang Q Musci T . Spatial and phenotypic heterogeneity of resident and monocyte-derived macrophages during inflammatory exacerbations leading to pulmonary fibrosis. Front Immunol. (2024) 15:1425466. doi: 10.3389/fimmu.2024.1425466 39100672 PMC11294112

[75] Li M Li J Wang Y Jiang G Jiang H Li M . Umbilical cord-derived mesenchymal stem cells preferentially modulate macrophages to alleviate pulmonary fibrosis. Stem Cell Res Ther. (2024) 15:475. doi: 10.1186/s13287-024-04091-7 39696548 PMC11657361

[76] Rahman M Wang ZY Li JX Xu HW Wang R Wu Q . Single-cell RNA sequencing reveals the interaction of injected ADSCs with lung-originated cells in mouse pulmonary fibrosis. Stem Cells Int. (2022) 2022:9483166. doi: 10.1155/2022/9483166 35450342 PMC9017459

[77] Ramachandran P Dobie R Wilson-Kanamori JR Dora EF Henderson BEP Luu NT . Resolving the fibrotic niche of human liver cirrhosis at single-cell level. Nature. (2019) 575:512–8. doi: 10.1038/s41586-019-1631-3 31597160 PMC6876711

[78] Kim SH Lee KY Chang K . The protective role of TREM2 in the heterogenous population of macrophages during post-myocardial infarction inflammation. Int J Mol Sci. (2023) 24 (6):5556. doi: 10.3390/ijms24065556 36982629 PMC10051125

[79] Wu X Pan G Chang L Liu Q Liu Y Zhang W . Endoplasmic reticulum stress-induced triggering receptor expressed on myeloid cells 2 (TREM2) downregulation exacerbates platelet activation and myocardial infarction in patients with coronary artery disease. J Am Heart Assoc. (2025) 14:e041220. doi: 10.1161/jaha.124.041220 40551335 PMC12450015

[80] Fu C Xu Q Liu J Tang S Liu C Cao Y . Triggering receptor expressed on myeloid cells-2 promotes survival of cardiomyocytes after myocardial ischemic injury through PI3K/AKT pathway. Cardiovasc Diagnosis Ther. (2022) 12:24–36. doi: 10.21037/cdt-21-490 35282669 PMC8898683

[81] Liu W Weng S Liu H Cao C Wang S Wu S . Serum soluble TREM2 is an independent biomarker associated with coronary heart disease. Clinica Chimica Acta; Int J Clin Chem. (2023) 548:117499. doi: 10.1016/j.cca.2023.117499 37536519

[82] Liu M Zhou X Wang Y Zhao W Zhao X Li L . A strategy involving microporous microneedles integrated with CAR-TREM2-macrophages for scar management by regulating fibrotic microenvironment. Advanced Materials (Deerfield Beach Fla). (2024) 36:e2406153. doi: 10.1002/adma.202406153 39313983

[83] Cao Y Wang Y Peng N Xiao J Wang S Fu C . The ratio of urinary TREM-1/TREM-2 mRNA expression in chronic kidney disease and renal fibrosis. Ann Med. (2021) 53:1010–8. doi: 10.1080/07853890.2021.1912384 34176389 PMC8245072

[84] Lu YP Wu HW Zhu T Li XT Zuo J Hasan AA . Empagliflozin reduces kidney fibrosis and improves kidney function by alternative macrophage activation in rats with 5/6-nephrectomy. Biomedicine Pharmacotherapy. (2022) 156:113947. doi: 10.1016/j.biopha.2022.113947 36411661

[85] Xiao Z Wang Y Chen Y Jin L Shi Y Liu C . Exosomes derived from TREM-2 knocked-out macrophages alleviated renal fibrosis via HSPa1b/AKT pathway. Am J Physiol Renal Physiol. (2025) 328:F131–51. doi: 10.1152/ajprenal.00219.2024 39657110

[86] Stephenson HG Betthauser TJ Jonaitis E Hulle V Kollmorgen G Quijano-Rubio C . Higher CSF sTREM2 is related to slower hippocampal atrophy and cognitive decline independently of pTau181 levels. Brain Behavior Immun. (2026) 134:106468. doi: 10.1016/j.bbi.2026.106468 41605308

[87] Wang R Zhan Y Zhu W Yang Q Pei J . Association of soluble TREM2 with Alzheimer's disease and mild cognitive impairment: a systematic review and meta-analysis. Front Aging Neurosci. (2024) 16:1407980. doi: 10.3389/fnagi.2024.1407980 38841103 PMC11150578

[88] Wilson EN Swarovski MS Linortner P Shahid M Zuckerman AJ Wang Q . Soluble TREM2 is elevated in Parkinson's disease subgroups with increased CSF tau. Brain: A J Neurol. (2020) 143:932–43. doi: 10.1093/brain/awaa021 32065223 PMC7089668

[89] Jiao L Yang J Wang W Liu X Fu Y Fan D . sTREM2 cerebrospinal fluid levels are a potential biomarker in amyotrophic lateral sclerosis and associate with UMN burden. Front Neurol. (2024) 15:1515252. doi: 10.3389/fneur.2024.1515252 39722698 PMC11669252

[90] Maeda T Mizutani Y Ohdake R Nagao R Kawabata K Shima S . Elevated levels of cerebrospinal fluid soluble triggering receptor expressed on myeloid cells 2 in multiple system atrophy: a marker of disease-associated microglial activation. J Neural Transm (Vienna Austria: 1996). (2026) 133:485–94. doi: 10.1007/s00702-025-03022-x 40986035 PMC12999635

[91] Guo T Ma J Sun J Xu W Cong H Wei Y . Soluble TREM2 is a potential biomarker for the severity of primary angiitis of the CNS. Front Immunol. (2022) 13:963373. doi: 10.3389/fimmu.2022.963373 36636326 PMC9831656

[92] Cuciuc V Tshori S Grib L Sella G Tuvali O Volodarsky I . Circulating soluble TREM2 and cardiovascular outcome in cohort study of coronary atherosclerosis patients. Int J Mol Sci. (2022) 23 (21):13121. doi: 10.3390/ijms232113121 36361908 PMC9656572

[93] Santol J Rajcic D Ortmayr G Hoebinger C Baranovskyi TP Rumpf B . Soluble TREM2 reflects liver fibrosis status and predicts postoperative liver dysfunction after liver surgery. JHEP Reports: Innovation Hepatol. (2025) 7:101226. doi: 10.1016/j.jhepr.2024.101226 40124168 PMC11929072

[94] Byers DE Wu K Dang-Vu G Jin X Agapov E Zhang X . Triggering receptor expressed on myeloid cells-2 expression tracks with M2-like macrophage activity and disease severity in COPD. Chest. (2018) 153:77–86. doi: 10.1016/j.chest.2017.09.044 29017955 PMC5812763

[95] Schauer SP Cho CH Novikova G Roth GA Lee J Sharma AD . Primate cerebrospinal fluid CHI3L1 reflects brain TREM2 agonism. Alzheimer's Dementia: J Alzheimer's Assoc. (2024) 20:5861–88. doi: 10.1002/alz.13921 39090679 PMC11497760

[96] Sun H Feng J Tang L . Function of TREM1 and TREM2 in liver-related diseases. Cells. (2020) 9 (12):2626. doi: 10.3390/cells9122626 33297569 PMC7762355

[97] Liu W Liu Y Guo X Cao C Weng S Peng D . The TREM receptor family in cardiovascular diseases: functions, mechanisms and therapeutic perspectives. Int Immunopharmacol. (2026) 172:116167. doi: 10.1016/j.intimp.2026.116167 41539000

[98] Colonna M Holtzman DM . Rethinking TREM2 as a target for Alzheimer's disease after the INVOKE-2 trial failure. Nat Med. (2025) 31 (10):3217–18. doi: 10.1038/s41591-025-03816-2 40603729

[99] Ma YN Hu X Karako K Song P Tang W Xia Y . The potential and challenges of TREM2-targeted therapy in Alzheimer's disease: insights from the INVOKE-2 study. Front Aging Neurosci. (2025) 17:1576020. doi: 10.3389/fnagi.2025.1576020 40353063 PMC12061918

[100] Ashvin Dhapola R Kumari S Sharma P Vellingiri B Medhi B . Unraveling the immune puzzle: role of immunomodulation in Alzheimer's disease. J Neuroimmune Pharmacology: Off J Soc NeuroImmune Pharmacol. (2025) 20:47. doi: 10.1007/s11481-025-10210-9 40299221

[101] Meier A Papapetropoulos S Marsh A Neelon K Stiles D O'Mara R . Phase 1, first-in-human, single-/multiple-ascending dose study of iluzanebart in healthy volunteers. Ann Clin Transl Neurol. (2025) 12:1065–76. doi: 10.1002/acn3.70033 40166927 PMC12093347

[102] Stangel M FD Shimshek D Gasparini F Galimberti I George N . VHB937, a TREM2 stabilizing and activating antibody strongly reduces pathology after peripheral administration in a broad range of animal models for neuroinflammation and neurodegeneration. Neurology. (2024) (7_supplement_1):5160. doi: 10.1212/wnl.0000000000205610

[103] Xie M Liu YU Zhao S Zhang L Bosco DB Pang YP . TREM2 interacts with TDP-43 and mediates microglial neuroprotection against TDP-43-related neurodegeneration. Nat Neurosci. (2022) 25:26–38. doi: 10.1038/s41593-021-00975-6 34916658 PMC8741737

[104] Trialists grapple with how to outsmart TREM2 (2025). Available online at: https://www.alzforum.org/news/conference-coverage (Accessed 17 April 2025).

[105] Park HJ Yang MJ Oh JH Yang YS Kwon MS Song CW . Genome-wide transcriptional response during the development of bleomycin-induced pulmonary fibrosis in sprague-dawley rats. Toxicol Res. (2010) 26:137–47. doi: 10.5487/tr.2010.26.2.137 24278517 PMC3834473

[106] Mahmoudian R Clavero-Mestres H Rusu EC Arredondo-Prats V Aguilar C Chicote JU . sTREM2 evaluation in women with metabolic dysfunction-associated steatotic liver disease: Advancing diagnostic approaches for early MASH. Eur J Internal Med. (2025) 141:106418. doi: 10.1016/j.ejim.2025.07.014 40716976

[107] Yu L Gang X Wang J Huang G Li Q Gu W . TREM2(+) macrophages accumulate in childhood IgA nephropathy and soluble TREM2 represents a reliable non-invasive biomarker. Exp Physiol. (2025). doi: 10.1113/ep092716 40321057 PMC13394584

